# A survey of morphological, molecular, and histopathological characteristics of *Taenia pisiformis* metacestode in Egyptian rabbits (*Oryctolagus cuniculus*)

**DOI:** 10.3389/fvets.2025.1701083

**Published:** 2025-12-05

**Authors:** Refaat Ras, Amanallah El-Bahrawy, Adel Abdelkhalek, Florica Morariu, Ayman N. Elsayed, Doaa S. Nouh, Anamaria Plesko, Marius Stelian Ilie, Manar AbdelMageed

**Affiliations:** 1 Department of Microbiology and Parasitology, Faculty of Veterinary Medicine, Badr University in Cairo (BUC), Badr City, Cairo, Egypt; 2 Department of Parasitology, Faculty of Veterinary Medicine, Zagazig University, Zagazig, Egypt; 3 Department of Pathology, Faculty of Veterinary Medicine, University of Sadat City, Sadat City, Minoufyia, Egypt; 4 Department of Food Hygiene, Safety and Technology, Faculty of Veterinary Medicine, Badr University in Cairo (BUC), Badr City, Cairo, Egypt; 5 Department of Biotechnologies, University of Life Sciences “King Mihai I” from Timișoara, Timişoara, Romania; 6 Department of Zoology and Entomology, Faculty of Science (Boys), Al-Azhar University, Nasr City, Cairo, Egypt; 7 Department of Anatomy and Embryology, Faculty of Veterinary Medicine, Zagazig University, Zagazig, Egypt; 8 Department of Parasitology and Parasitic Disease, Faculty of Veterinary Medicine, University of Life Sciences “King Mihai I” from Timișoara, Timișoara, Romania; 9 Department of Pathology and Clinical Pathology, Faculty of Veterinary Medicine, Badr University in Cairo (BUC), Badr City, Cairo, Egypt; 10 Department of Pathology, Faculty of Veterinary Medicine, Zagazig University, Zagazig, Egypt

**Keywords:** cysticercosis, *pisiformis*, rabbits, prevalence, molecular

## Abstract

**Introduction:**

*Taenia pisiformis (T. pisiformis)*, the etiological agent of cysticercosis in rabbits, poses a considerable health risk to domestic lagomorphs and contributes to economic losses in rabbit farming. This study aimed to ascertain the prevalence, risk factors, and molecular characteristics of *T. pisiformis* in rabbits from three Egyptian regions: Badr City (Cairo Province), Sadat City (Monufia Province), and Assiut City (Assiut Province).

**Methods:**

A total of 150 samples were collected from both home-raised (*n* = 77) and farm-raised (*n* = 73) rabbits from January 2024 to December 2024. *T. pisiformis* cysts were identified morphologically and histologically, with tissue samples processed using hematoxylin and eosin (H&E) staining. Molecular confirmation was performed using polymerase chain reaction (PCR) targeting the cytochrome oxidase subunit 1 (*cox1*) and NADH dehydrogenase subunit 1 (*nad1*) mitochondrial genes, followed by sequencing and phylogenetic analysis. Statistical associations between infection and risk factors (age, location, season, and management system) were evaluated using the chi-square test and Fisher’s exact test.

**Results:**

The overall infection prevalence rate was 21.3% (32/150), which was significantly higher in home-raised rabbits (31/77, 40.2%) than in farm-raised rabbits (1/73, 1.3%; χ^2^ = 31.5, *p* < 0.001). Infection rates were also strongly linked to season, with fall and winter showing higher prevalence. Cysts were mostly found in the mesentery and varied in number (1–5 per rabbit). Morphologically, the cysts contained a scolex with distinctive features, including suckers and rostellar hooks. Histology showed a thick cyst wall and characteristic tissue structures. Molecular analysis confirmed the parasite as *T. pisiformis*, with sequence similarities ranging from 97.64 to 100%, indicating a close relationship to global sequences.

**Discussion:**

These findings underscore the influence of management practices and seasonal factors on infection dynamics and highlight the importance of molecular tools in parasite surveillance.

## 1 Introduction

*Cysticercus pisiformis (C. pisiformis)*, the larval form of *Taenia pisiformis (T. pisiformis)*, is the most frequent cestode and is widely distributed worldwide, primarily affecting rabbits and hares (1). The lifecycle involves canids, specifically dogs and foxes, as definitive hosts. Rabbits acquire infection through the infection of feed contaminated with eggs shed in the feces of these definitive hosts (2). Following ingestion, the eggs hatch, and the hexacanth embryos subsequently penetrate the intestinal wall. Then, they migrate through the portal vein and differentiate into the larval stage or metacestode, specifically *C. pisiformis*, which appears as a fluid-filled cyst characterized by an armed scolex (3).

In Egypt, the majority of rabbit production (approximately 57%) is carried out by rural families who typically raise small flocks (4). Severe larval infection can result in hepatic damage in intermediate hosts, potentially leading to hepatitis, cirrhosis, gastrointestinal disturbances, and impaired immune responses. Consequently, this can lead to secondary bacterial infection and economic losses in the rabbit breeding industry due to their deleterious effects on rabbit health and productivity (5–7). Moreover, *T. pisiformis* infestation affects the behavioral and productive characteristics of rabbits, and obesity exacerbates the consequences of infection (8). In addition, infection reduces fecundity in rabbits, with infected does showing decreased embryo implantation and smaller embryo vesicle sizes compared to uninfected does (9). However, no zoonotic cases of *C. pisiformis* were recorded (10), but potential risks should be considered for future studies.

Several studies have reported cysticercosis in rabbits. In southern Spain, a study of 2,923 wild rabbits reported an overall prevalence of 2.8% (6). In contrast, a study in Poland reported a significantly higher prevalence of gastrointestinal parasites of 79.56% in slaughtered rabbits, with *T. pisiformis* being specifically found in 4.74% of the examined animals (11). Furthermore, in Mexico, the first formal report of infection with *T. pisiformis* metacestodes revealed a prevalence of approximately 70% (12). Although prevalence studies offer valuable insights into the geographical distribution and burden of *T. pisiformis*, a more comprehensive understanding of the parasite’s biology and epidemiology requires molecular approaches, particularly genetic characterization (13). Mitochondrial DNA is a highly powerful and reliable molecular marker for analyzing population structure and evolutionary history (14). The mitochondrial genes, such as cytochrome oxidase subunit 1 (*cox1*) and NADH dehydrogenase subunit 1 (*nad1*), have been widely used to investigate the genetics of taeniid cestodes, including their genetic origins, population boundaries, and intraspecific and interspecific variation (15–17).

Hence, in Egypt, raising rabbits in small backyard colonies has traditionally served as a means of supplementing household income. However, this practice has recently evolved into a distinct source of meat production. Rabbits are considered optimal animals for meat production because of their quick feed-to-meat conversion, short lifespan, and short pregnancy period (18, 19). With the increasing importance of rabbit farming as a commercial meat source, the impact of parasitic diseases such as *T. pisiformis* on productivity and animal welfare is becoming a growing concern. Although *T. pisiformis* is one of the most prevalent parasites that severely affect rabbit breeding in Egypt, there are no formal reports on the infection of rabbit populations with *T. pisiformis* metacestodes. There is only one study that was conducted in the Qena Governorate, Egypt, which identified *T. pisiformis* in domestic rabbits (20).

In addition to this isolated case, data on the prevalence and distribution of *T. pisiformis* in Egypt remain limited and geographically restricted, offering no nationwide perspective on its impact. Furthermore, the genetic variability of circulating *T. pisiformis* strains in Egypt has never been characterized using molecular tools, despite the value of such information in understanding transmission routes and informing targeted control measures. The lack of effective vaccines and antiparasitic medications has resulted in the poor control of parasitic infections at present. Consequently, integrating molecular diagnostics into routine surveillance could significantly enhance disease management in rabbit production systems.

However, to date, no studies have utilized mitochondrial gene markers to characterize *T. pisiformis* strains in Egypt, representing a significant gap in the molecular epidemiology of this parasite in the region.

Therefore, this study aimed to investigate the prevalence, determine the morphological and histopathological characteristics, and conduct a phylogenetic analysis of *T. pisiformis* in domestic rabbits in Egypt, using both traditional and molecular tools.

## 2 Materials and methods

### 2.1 Ethical approval

All procedures conducted in this study were approved by the Research Ethics Committee at the Faculty of Pharmacy, Badr University in Cairo, Cairo, Egypt (Protocol No. BUC-IACUC/VET/161/A/2024).

### 2.2 Sample collection

In this study, a total of 150 domestic rabbits were collected from three different locations: Badr City (Cairo Province), Sadat City (Monufia Province), and Assiut (Assiut Province). The examined animals included rabbits that had died from illness and were submitted for diagnostic evaluation, as well as apparently healthy rabbits reared within the same localities. All rabbits were consecutively received at the Department of Pathology and Clinical Pathology, School of Veterinary Medicine, Badr University in Cairo, Egypt, between January and December 2024. To ensure the reliability of parasitological and pathological examination, carcasses presented more than 12 h postmortem were excluded. Apparently healthy rabbits, which were submitted by veterinarians or owners due to suspected herd health concerns and as part of diagnostic investigations within the affected localities, were humanely euthanized to allow a thorough examination of internal parasites and associated lesions. Euthanasia was conducted in full accordance with international and institutional ethical guidelines using intravenous administration of an overdose of sodium pentobarbital (100 mg/kg). Death was confirmed by the absence of vital signs prior to decapitation.

During necropsy, a thorough examination of all organs was performed, and cysts indicative of *T. pisiformis* were carefully extracted and subjected to both macroscopic and microscopic morphological evaluation. Data on management systems were recorded, classifying rabbits as either home-raised (small-scale backyard rearing) or farm-raised (small-scale commercial farming). Age was also documented and considered a potential risk factor in epidemiological analysis.

### 2.3 Histological examination

Tissue specimens of suspected *T. pisiformis* cysts, liver, and other organ tissues were collected from necropsied rabbits and immediately fixed in 10% neutral-buffered formalin after which they were sequentially processed for the preparation of paraffin blocks. Subsequently, the blocks were cut into 4–5 μm thin sections and stained with hematoxylin and eosin (H&E) for histopathological assessment (21). Photomicrographs were obtained using a light microscope equipped with an AmScope MU1803-HS microscope digital camera.

### 2.4 Molecular identification of identified *Cysticercus pisiformis*

#### 2.4.1 DNA extraction

DNA was extracted from seven cyst-positive isolates from tissues using the DNeasy Blood & Tissue Mini-Kit (Qiagen, Hilden, Germany) following the manufacturer’s recommendations (22, 23). Briefly, 25 mg of tissue was sectioned into small fragments and incubated with 20 μL of Proteinase K at 56 °C until complete tissue lysis occurred. Subsequently, 200 μL of AL buffer and absolute ethyl alcohol were added sequentially and homogenized via vortexing. The resulting homogeneous solution was then transferred to a silica column and centrifuged. Subsequently, the samples were purified and centrifuged according to the manufacturer’s protocol. The eluted DNA was stored at −20 °C for further analysis.

#### 2.4.2 DNA amplification by PCR

Extracted DNA was amplified by conventional PCR using two specific primer sets targeting the *cox1* and *nad1* mitochondrial genes. For *cox1*, amplification was performed using the forward primer JB3 (5′-TTTTTTGGGCATCCTGAGGTTTAT −3′) and the reverse primer JB4.5 (5′-TAAAGAAAGAACATAATGAAAATG-3′) (24). The second set of primers targeting the *nad1* gene were forward primer JP11 (5′-AGATTCGTAAGGGGCCTAATA-3′) and reverse primer JP12 (5′-ACCACTAACTAATTCACTTTC-3′) (25). Each PCR reaction was carried out in a total volume of 50 μL of mixture containing 25 μL of GeneDireX OnePCR™ Master Mix (Cat# MB203-0050), 1 μL of DNA template, 1 μL of each 10 μM the forward primer and the reverse primer, and 22 μL of nuclease-free water. The cycling conditions of PCR were the same for both genes and were set up as follows: an initial denaturation for 5 min at 94 °C, followed by 35 cycles of denaturation at 94 °C for 40 s, annealing at 50 °C for 1 min, extension at 72 °C for 2 min, and a final extension step at 72 °C for 5 min. The reactions were performed on a gradient thermal cycler (Benchmark, Thermal Cycler, United States). The amplicons, as well as a 100 bp ladder (Cat# DM003-R500), were separated on 1.5% agarose gel containing 0.4 μg/mL of ethidium bromide in 1x TAE buffer and visualized under a UV transilluminator. To ensure data reliability, *Cysticercus tenuicollis* DNA (previously confirmed in our earlier studies) was used as a positive control, and nuclease-free water was used as a negative control in all PCR runs. All amplification steps were performed in separate pre- and post-PCR areas to minimize contamination.

#### 2.4.3 Sample selection and sequencing

Although multiple samples yielded successful amplification for both mitochondrial markers, four representative isolates per gene were selected for sequencing based on the intensity and clarity of their PCR bands, as well as to ensure geographic representation from the three collection sites (Badr, Menoufia, and Assiut). The purified PCR products were sequenced in the forward direction using the same forward primers and the Sanger dideoxy method (Macrogen Inc., Seoul, South Korea; https://dna.macrogen.com).

Chromatograms were visually inspected to confirm base-calling accuracy. All sequences showed clear, high-quality peaks with no ambiguous positions, and no “N” symbols were required in the final sequences. Low-quality ends (Phred <20) were trimmed prior to alignment. The resulting sequences had no unresolved bases and were directly used for BLAST comparison on the NCBI database and phylogenetic analysis. The sequences of the current study were deposited in the GenBank database with available data.

#### 2.4.4 Sequencing and phylogenetic analysis

Multiple sequence alignment of the *cox1* and *nad1* sequences with related *Taenia* species retrieved from the GenBank database was performed using Clustal W and Clustal X v2.0 (26). Alignments were visualized and manually adjusted using Jalview v2.11.0 (27). Evolutionary relationships were inferred using both the neighbor-joining (NJ) and maximum likelihood (ML) methods implemented in MEGA 11 (28). For NJ analysis, evolutionary distances were computed using the maximum composite likelihood method (29) with 1,000 bootstrap replicates to assess branch support (30). For ML analysis, the best-fit substitution models were determined using the model-test option in MEGA 11: HKY + G + I for *cox1* and HKY + G for *nad1* (31). ML trees were reconstructed with 1,000 bootstrap replicates, and the resulting bootstrap consensus trees were used for interpretation (30).

*Diphyllobothrium* sp. was selected as the outgroup to root all phylogenetic trees. Bootstrap values were displayed next to the corresponding nodes.

#### 2.4.5 Quality control and data validation

All molecular analyses were performed under strict quality control conditions. Separate workstations were used for DNA extraction, PCR setup, and post-PCR analysis to avoid cross-contamination.

Each sequence chromatogram was reviewed to confirm peak clarity and exclude sequencing artifacts. Trimming, alignment, and phylogenetic procedures were performed consistently across all samples to ensure reproducibility and comparability between the *cox1* and *nad1* datasets.

### 2.5 Statistical analysis

Statistical analyses were conducted using R 4.4.1 software (R Foundation for Statistical Computing, Vienna, Austria). Descriptive statistics were computed for infection status as the outcome and risk factors, respectively. Independent variables of interest were the age at sample collection (2, 3, or 4 months), management systems (home or farm), sites of collection Badr City (Cairo), Sadat City (Monufia), or Assiut City (Assiut), and seasons of collection (fall, winter, or spring). To investigate the association between these risk factors and the outcome, a chi-square test was used, and statistical significance was set at a *p-*value of < 0.05. When the assumptions of the chi-square test were not met, Fisher’s exact test was used instead. To determine which specific groups differed significantly when the overall test indicated a significant result, standardized residuals were calculated to measure the difference between the observed and expected frequencies for each cell (32). There is a significant deviation whenever residuals are greater than 1.96.

## 3 Results

### 3.1 Descriptive statistics

A total of 150 samples were collected from either home-raised or farm-raised rabbits in Badr City (Cairo), Sadat City (Monufia), and Assiut City (Assiut Province). Of those samples, 32 (21.3%) tested positive for the presence of *T. pisiformis*. One cyst was isolated from 17 (53%) of the positive samples, two cysts were isolated from 8 (25%) of the samples, three cysts were recovered from 5 (16%) of the samples, and four cysts were isolated from one sample, and five cysts from another one sample, which together accounted for 3% of the samples.

**Figure 1:**
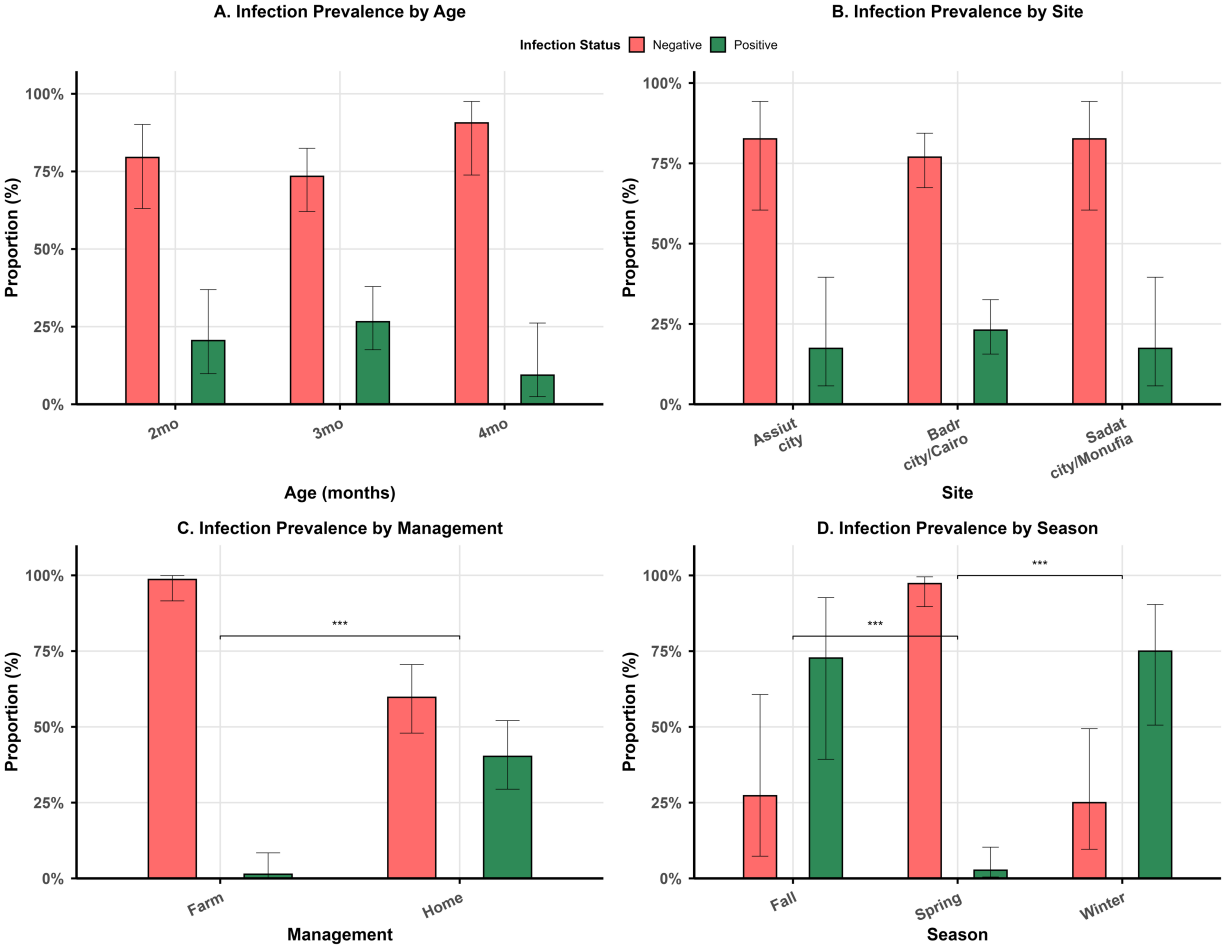
Bar plots show the distribution of positive versus negative samples by age **(A)**, sites of sample collection **(B)**, management systems **(C)**, and season **(D)**. Asterisk *** indicates significant difference in **(C,D)**.

Of the 150 samples, 77 (51.3%) were collected from home-raised rabbits, while the rest of the samples (73, 48.7%) were collected from farm-raised rabbits. Interestingly, only one sample tested positive from farm-raised rabbits (1/73, 1.3%), while 31 samples tested positive from home-raised (40.2%). More information on the distribution of positive and negative samples by age, sampling sites, management systems, and season is provided in Figures 1A–D and Table 1. However, data on seasonal variation were available for only 105 samples.

**Table 1:** Prevalence of *T. pisiformis* in relation to various risk factors.

Variable	No. examined	No. positive	Prevalence % (positive/examined)
Site			
Assiut City	23	4	17.4
Sadat City	23	4	17.4
Badr City	104	24	23.1
Age			
2 months	39	8	20.5
3 months	79	21	26.6
4 months	32	3	9.4
Management			
Farm	73	1	1.4
Home	77	31	40.2
Season*			
Fall	11	8	72.7
Winter	20	15	75
Spring	74	2	2.7

* The seasonal information was available for 105 samples.

### 3.2 Association between infection status and risk factors

Management was significantly associated with the infection status (X^2^ = 31.5, *p* < 0.001). Standardized residuals of 5.8 indicated that farm-raised rabbits were more likely to have negative samples, whereas the corresponding residuals for home-raised rabbits indicated its association with positive samples for *T. pisiformis*. Furthermore, the season at sample collection was significantly associated with *T. pisiformis* infection (*p* < 0.001), where residuals of −3.7 indicated that samples collected in spring were more likely to be negative in contrast to residuals of 3.3 and 4.6 for fall and winter, respectively, indicating their strong association with positive samples for *T. pisiformis*. On the other hand, neither the age of the animals nor the sites of sample collection were significantly associated with infection status (X^2^ = 4.03, *p* = 0.13 for age; *p* = 0.86 for sites).

### 3.3 Morphological characteristics

**Figure 2:**
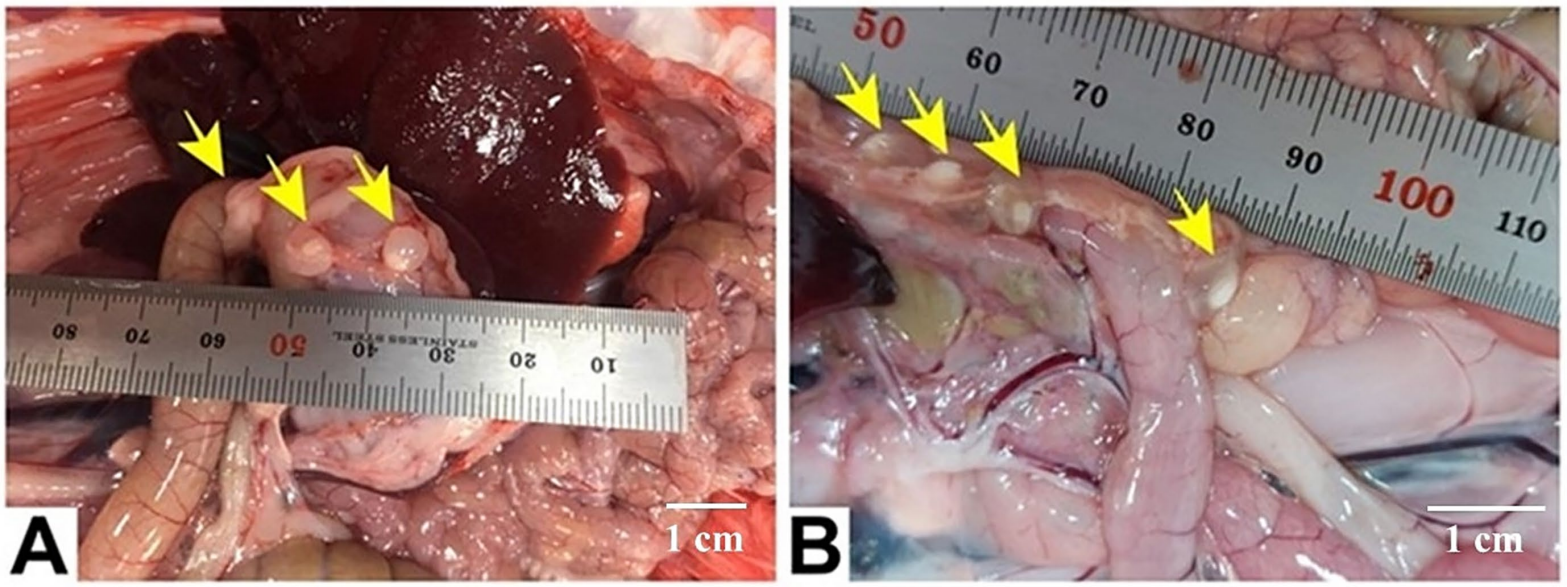
Gross appearance of *C. pisiformis* cysts (yellow arrows) represented by several small bladder cysts, each containing a single whitish scolex and clear fluid, attached to viscera in the peritoneal cavity near the stomach **(A)** and the intestine **(B)**. Scale bar = 1 cm in **(A,B)**.

**Figure 3:**
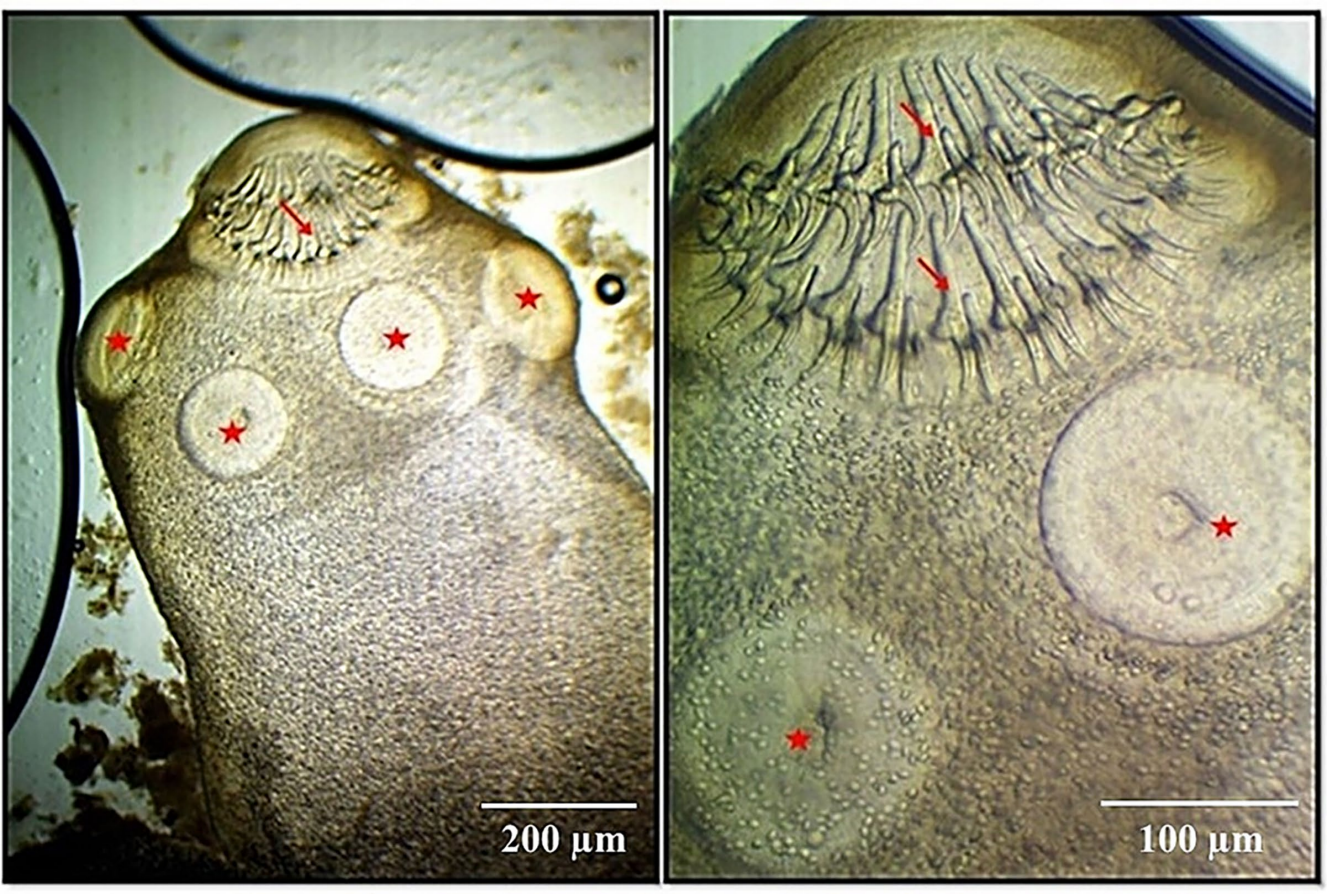
Micrographs of *C. pisiformis* scolex: the scolex has four suckers, and an armed rostellum has double rows of hooks. Suckers (star) and hooks (arrow). Scale bar = 200 um (not stained 4x (left)). Scale bar = 100 um (not stained 10x (right)).

The cystic stage of *T. pisiformis* appeared as a small, bladder-like structure measuring diameter of 5–8 mm, distinguished by a single invaginated scolex. The mature scolices displayed a substantial size, each bearing four prominent lateral suckers. The rostellum exhibited two distinct rows of hooks characteristic of Taeniidae morphology, consisting of a blade, handle, and guard structure. The total number of hooks was 36, organized into two rows with 18 large hooks and 18 small hooks. The large hooks measured between 112 and 201 μm in length, whereas the smaller hooks ranged from 65 to 121 μm. The suckers were spherical with thick muscular walls and displayed diameters between 96 and 188 μm (Figures 2, 3).

### 3.4 Histopathological findings

**Figure 4:**
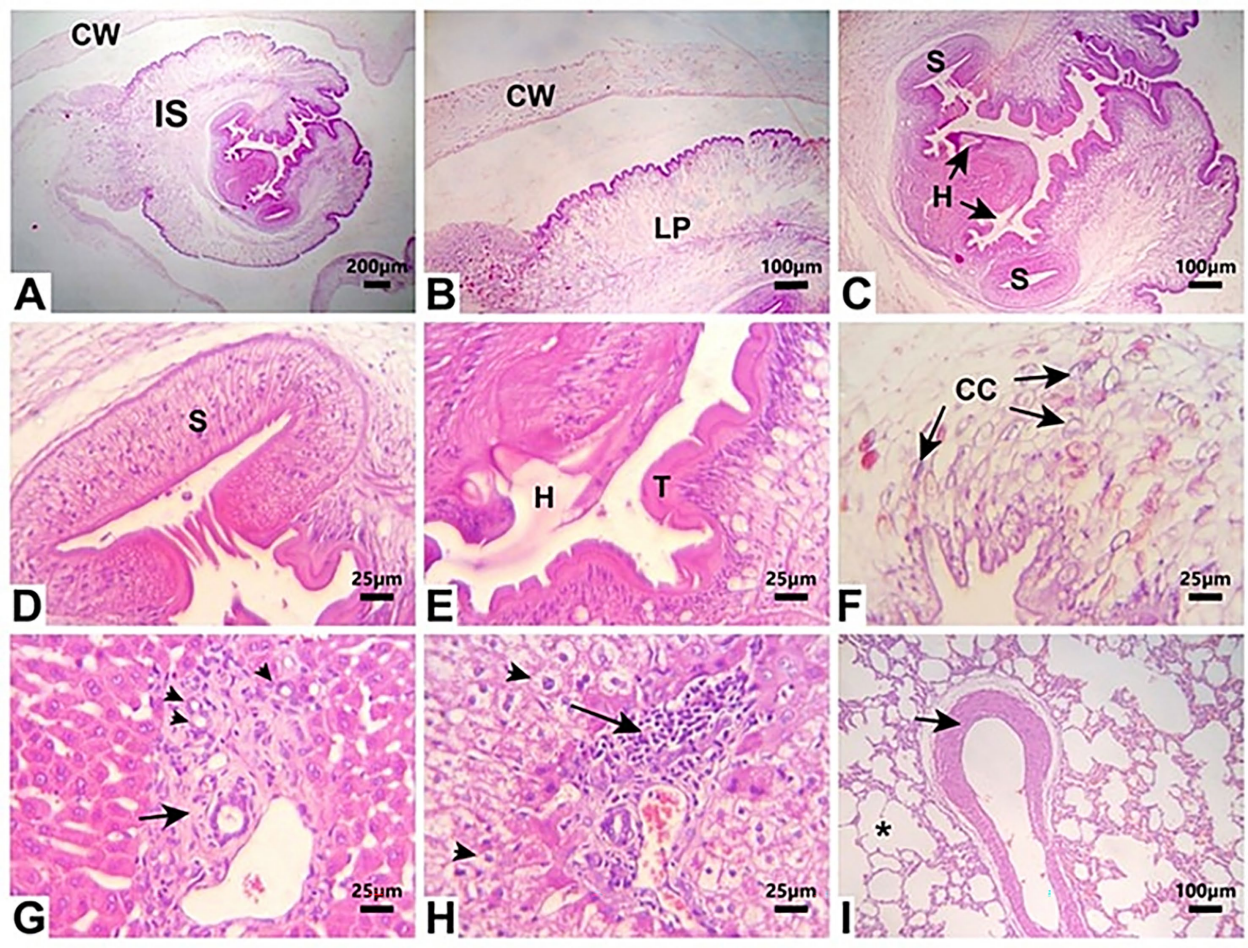
Photomicrographs of H&E-stained tissue sections from necropsied rabbits **(A–F)** *C. pisiformis* larval cyst shows a single invaginated scolex (IS) surrounded by a thick fibrous cyst wall (CW). The invaginated scolex displays suckers (S) and hooks **(H)**. The larval parenchyma (LP) is surrounded by a serrated cuticle (T) and contains numerous basophilic calcareous corpuscles (CC). **(G)** Liver tissue section with portal area shows proliferation of reactive bile ducts (arrowheads) and fibrous connective tissue proliferation (arrow). **(H)** Liver tissue section shows the vacuolation of hepatocytes (arrowheads) and moderate periportal lymphoplasmacytic infiltration. **(I)** Lung tissue section shows medial hypertrophy of a blood vessel (arrow) and mild emphysema (asterisk). Scale bar = 200 um in **(A)**. Scale bar = 100 um in **(B,C,I)**. Scale bar = 25 um in **(D–H)**.

Larval cysts of *C. pisiformis* in the peritoneal cavity exhibited a solitary invaginated scolex surrounded by a thick fibrous cyst wall. The invaginated scolex featured the typical suckers and hooks of cysticerci. The larval parenchyma was surrounded by the serrated cuticle and contained numerous basophilic calcareous corpuscles. The liver section showed bile ductular reaction and portal fibrous connective tissue proliferation. The lung section showed vascular medial hypertrophy and mild emphysema (Figure 4).

### 3.5 Molecular and phylogenetic analyses

#### 3.5.1 Molecular characterization

Several *T. pisiformis* cyst samples were successfully amplified using PCR, yielding distinct mitochondrial fragments of approximately 450 bp for the *cox1* gene and 500 bp for the *nad1* gene. From these, four representative isolates per gene, exhibiting strong and well-defined amplification profiles and originating from the three primary collection sites (Badr, Menoufia, and Assiut), were selected for sequencing.

**Figure 5:**
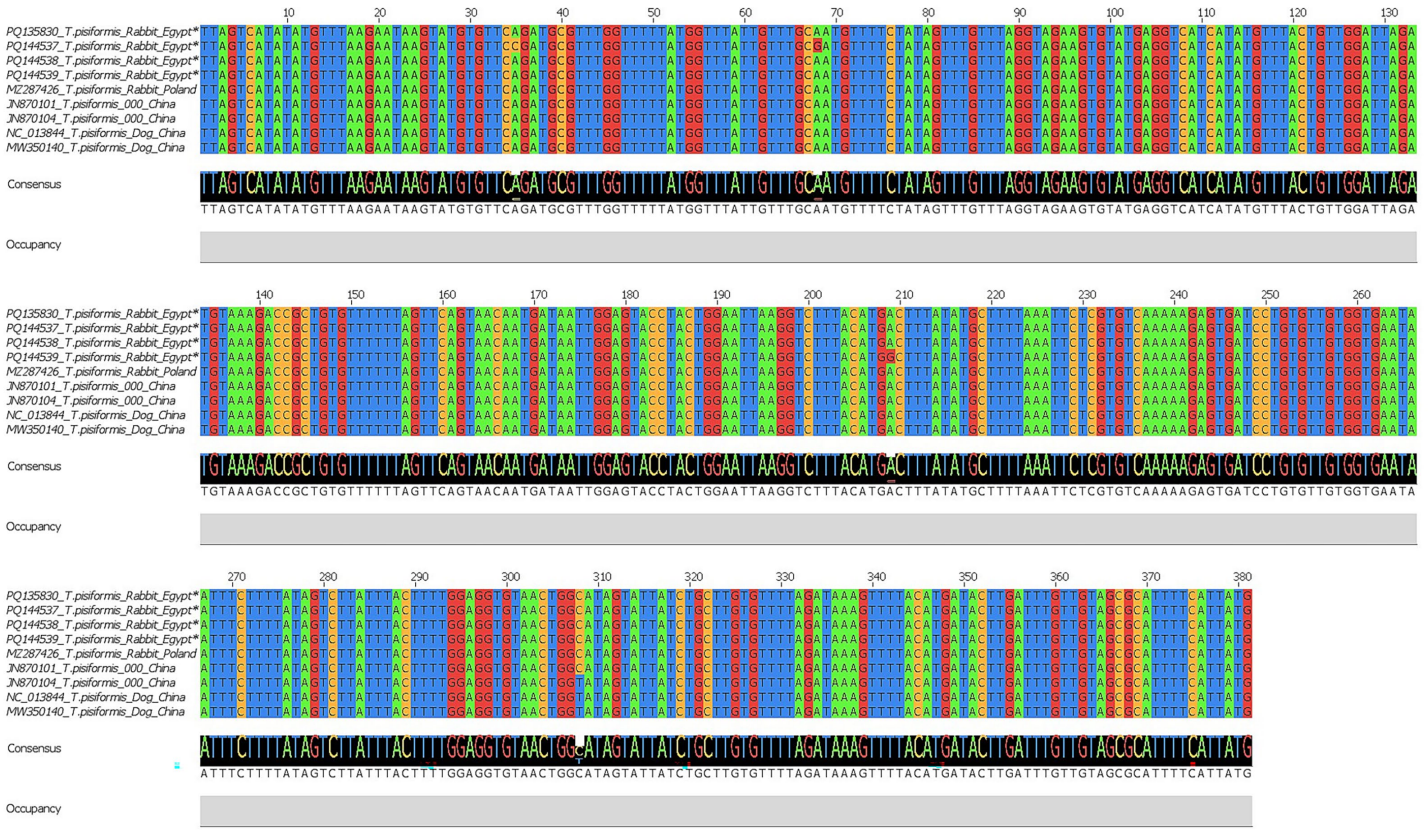
Multiple sequence alignment of the *cox1* gene of *T. pisiformis*. Sequences obtained in this study are indicated by asterisks (*). The alignment compares these sequences with homologous *T. pisiformis* sequences retrieved from the GenBank database.

For the *cox1* gene, four sequences were obtained and deposited in the GenBank database under accession numbers PQ135830, PQ144537, PQ144538, and PQ144539. BLAST analysis revealed 99.21–100% identity with *T. pisiformis* sequences from the GenBank database (query cover 100%), confirming accurate molecular identification. Multiple sequence alignment of the *cox1* sequences from Egyptian rabbit isolates, compared with global isolates from China and Poland, showed a high level of nucleotide conservation interspersed with variable positions. Several single-nucleotide polymorphisms (SNPs) were detected at positions 35, 68, 209, and 308, indicating low but measurable intraspecific variation among the isolates. These variations were visualized using Jalview v2.11.0, as shown in Figure 5.

Similarly, four *nad1* sequences were successfully obtained and registered in the GenBank database under accession numbers PQ156139–PQ156142. BLAST results demonstrated 97.64–100% identity with *T. pisiformis* isolates from rabbit and dog hosts (query cover 100%). The alignment of the *nad1* sequences from Egypt, China, and Poland revealed clear conservation across large regions, with variable sites detected at positions 37, 93, 102, 135, 136, 158, 216, 257, 303, 319, 330, 331, 345, 373, 429, and 458.

**Figure 6:**
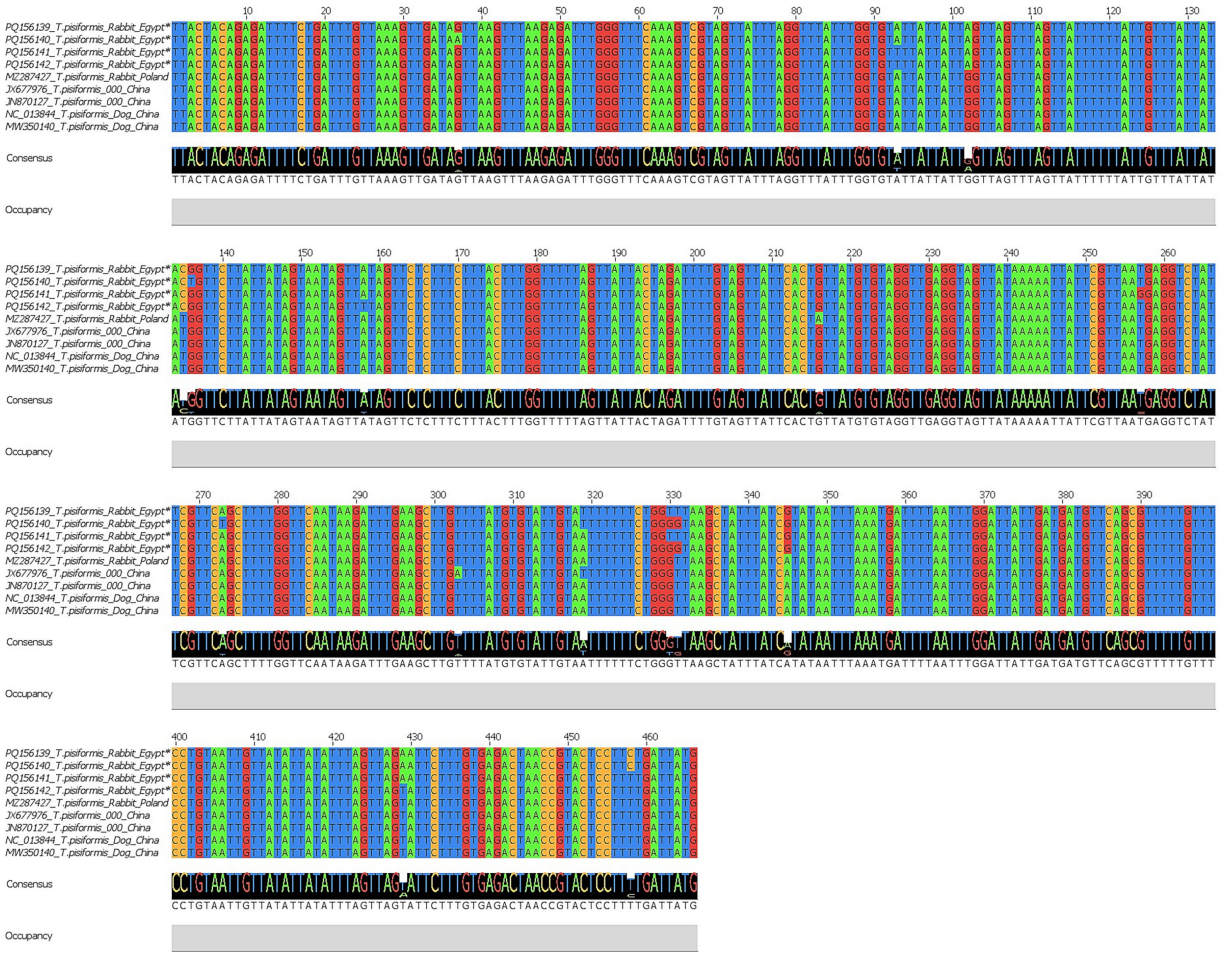
Multiple sequence alignment of the *nad1* gene of *T. pisiformis*. Sequences obtained in this study are indicated by asterisks (*). The alignment compares these sequences with homologous *T. pisiformis* sequences retrieved from the GenBank database.

These polymorphisms suggest the presence of minor genetic variation potentially related to host or geographic origin, as shown by Jalview v2.11.0 in Figure 6.

#### 3.5.2 Phylogenetic analysis (neighbor-joining method)

Phylogenetic analyses were first performed using the neighbor-joining (NJ) method based on 19 *cox1* and 17 *nad1* nucleotide sequences. Ambiguous positions were removed from each sequence pair, resulting in final alignments of 318 and 327 positions, respectively. In both trees, the Egyptian isolates clustered within the *T. pisiformis* clade and showed minimal intraspecific variation.

**Figure 7:**
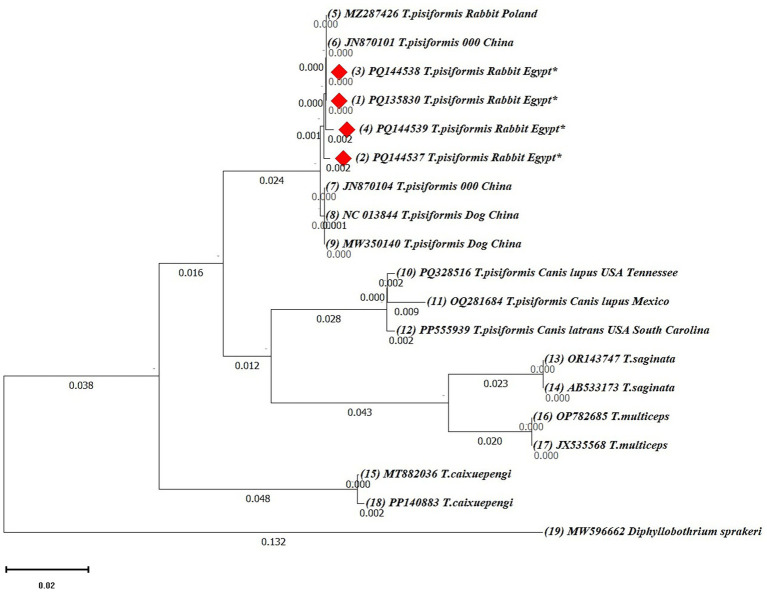
Neighbor-joining (NJ) phylogenetic tree of *cox1* gene sequences of *Taenia* species. The tree was constructed based on evolutionary distances computed using the maximum composite likelihood method. Branch lengths are drawn to scale, representing the number of substitutions per site. The tree is rooted with *Diphyllobothrium sprakeri* as the outgroup. Sequences from this study are marked with red diamonds (♦).

The *cox1*-based NJ tree grouped the Egyptian sequences closely with isolates from Poland (MZ287426) and China (NC013844, MW350140, JN870101, and JN870104), confirming their genetic identity and close evolutionary relationship (Figure 7). Additionally, sequences from wolves and coyotes (PQ328516, OQ281684, and PP555939) formed closely related but distinct subclusters, suggesting potential host-specific adaptation or early divergence. Other *Taenia* species, including *Taenia saginata* (OR143747, AB533173), *Taenia multiceps* (OP782685, JX535568), and *Taenia cakuepengi* (MT882036 and PP140883), formed distinct and separate clades, whereas *Diphyllobothrium sprakeri* (MW596662) served as the outgroup and was positioned on the most distant branch, confirming clear phylogenetic separation.

**Figure 8:**
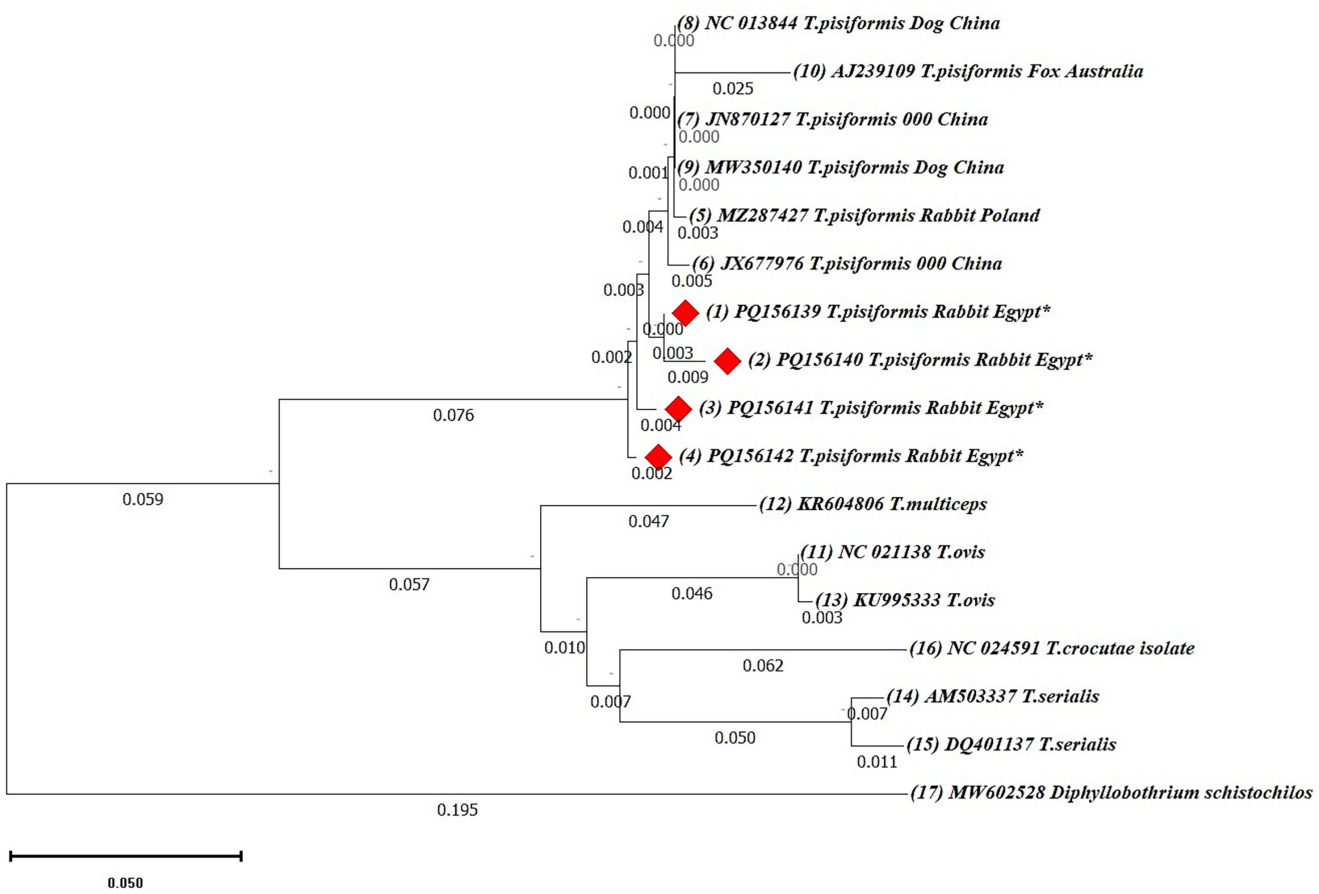
The neighbor-joining (NJ) phylogenetic tree of *nad1* gene sequences of *Taenia* species. The tree was constructed based on evolutionary distances computed using the maximum composite likelihood method. Branch lengths are drawn to scale, representing the number of substitutions per site. The tree is rooted with *Diphyllobothrium schistochilos* as the outgroup. Sequences from this study are marked with red diamonds (♦).

The *nad1*-based NJ tree also revealed a consistent topology, with the Egyptian *T. pisiformis* isolates clustering strongly with global sequences from rabbit and dog hosts, as well as a fox-derived isolate (AJ239109), which grouped within the clade but exhibited a slightly higher genetic distance, suggesting potential host-specific adaptation or early divergence (Figure 8). Other *Taenia* species, including *T. multiceps* (KR604806), *Taenia ovis* (NC021138, KU995333), *Taenia serialis* (AM503337, DQ410137), and *Taenia crocutae* (NC024591), formed distinct and separate clades, whereas *Diphyllobothrium schistochilos* (MW602528) served as the outgroup and was positioned on the most distant branch, confirming clear phylogenetic separation.

#### 3.5.3 Phylogenetic analysis (maximum likelihood method)

**Figure 9:**
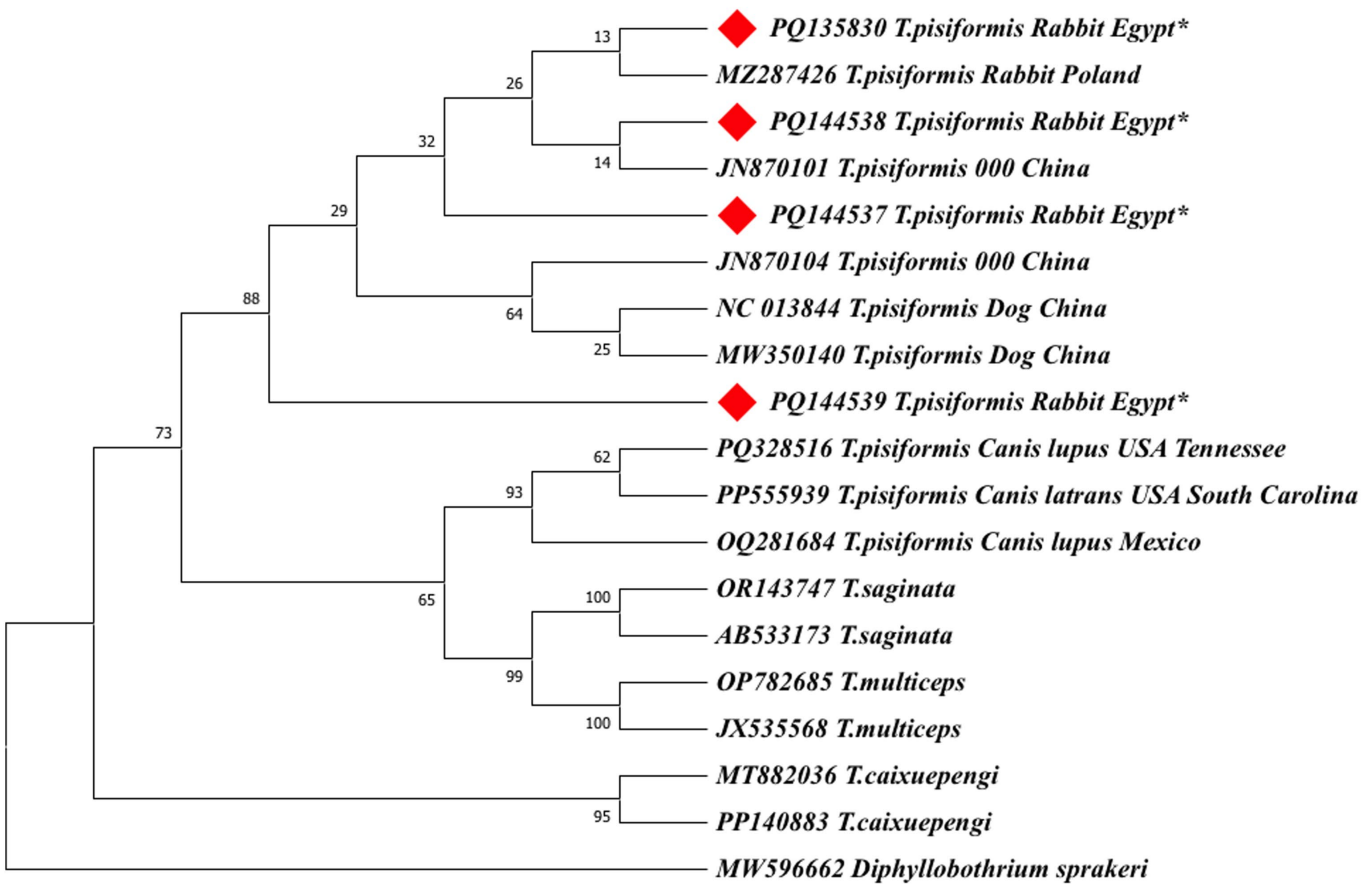
The maximum likelihood (ML) phylogenetic tree based on *cox1* gene sequences of *Taenia* species, inferred under the Hasegawa–Kishino–Yano (HKY) + G + I model. Bootstrap values (1,000 replicates) are shown at the nodes. Branch lengths are proportional to the number of substitutions per site. The tree was rooted with *Diphyllobothrium sprakeri*. Sequences from this study are marked with red diamonds (♦).

**Figure 10:**
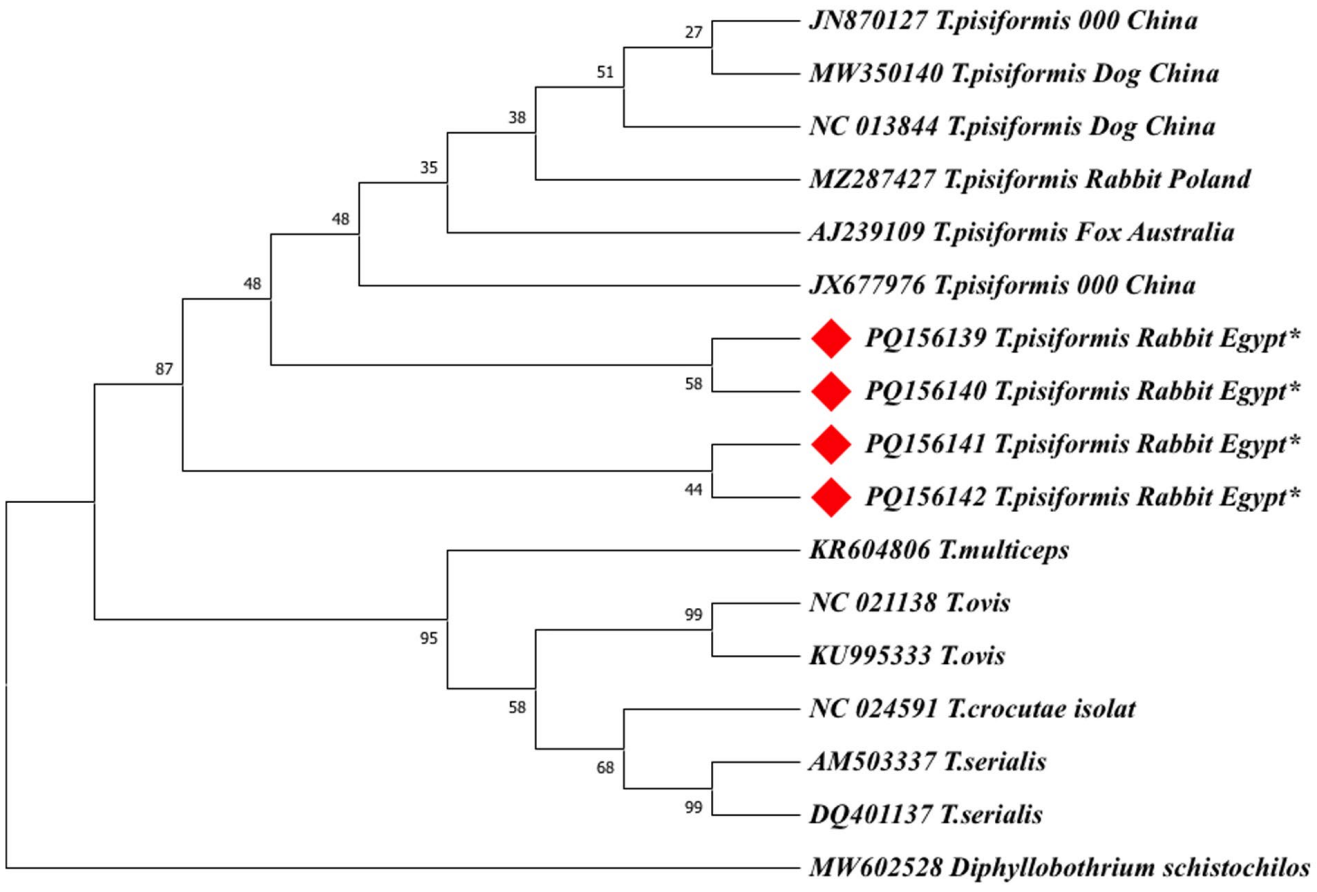
The maximum likelihood (ML) phylogenetic tree based on *nad1* gene sequences of *Taenia* species, inferred under the HKY + G model. Bootstrap values (1,000 replicates) are shown at the nodes. Branch lengths are proportional to the number of substitutions per site. The tree was rooted with *Diphyllobothrium schistochilos*. Sequences from this study are marked with red diamonds (♦).

To validate the NJ-based results, additional phylogenetic trees were constructed using the maximum likelihood (ML) method for both *cox1* and *nad1* genes (Figures 9, 10).

Both ML trees showed consistent topologies with the NJ trees, supporting the robustness of the phylogenetic inference.

In the *cox1*-based ML tree (Figure 9), the Egyptian isolates formed a well-supported clade (bootstrap values >70%) with other global *T. pisiformis* sequences from rabbits and dogs, confirming their genetic identity within the species. Sequences from wild canids again formed distinct subgroups, supporting the potential host-specific patterns observed in the NJ tree. Other *Taenia* species formed clearly separated clades, with *Diphyllobothrium sprakeri* as the outgroup.

In the *nad1*-based ML tree (Figure 10), the Egyptian isolates form a monophyletic cluster with strong support (bootstrap = 95%) within a larger clade containing global *T. pisiformis* sequences from rabbits, dogs, and a fox. The fox-derived isolate (AJ239109) grouped within the main *T. pisiformis* clade but showed a slightly separate position with weak nodal support (bootstrap = 35%), reinforcing the possibility of host-associated genetic variation. Other *Taenia* species formed distinct and well-separated clades with varying bootstrap support: *T. multiceps* (95%), *T. ovis* (58%), *T. serialis* (99%), and *T. crocutae* (68%). The clear separation of these congeneric species was further confirmed by the maximum support (bootstrap = 99%) for the deeper nodes defining their distinct lineages. *Diphyllobothrium schistochilos* served as the outgroup, occupying the most distant branch and confirming robust phylogenetic structure. These findings, despite the minor SNPs detected in the alignments, collectively affirm that *T. pisiformis* isolates from Egyptian rabbits are genetically homogeneous and closely related to *T. pisiformis* isolates from other geographic regions.

#### 3.5.4 Genetic distance estimation

The evolutionary divergence among *cox1* and *nad1* sequences of *Taenia* species and outgroup taxa was estimated using the maximum composite likelihood model in the MEGA11 software.

The average evolutionary divergence values were 0.0846 for *cox1* (with *D. sprakeri* as the outgroup) and 0.1512 for *nad1* (with *D. schistochilos* as the outgroup). These data, summarized in Supplementary Tables 1, 2, indicate moderate interspecific genetic distances between *T. pisiformis* and other *Taenia* species.

## 4 Discussion

*T. pisiformis*, one of the most common intestinal parasites in canines, significantly affects rabbit health and causes notable economic losses in the rabbit breeding industry (33). In Egypt, research on *T. pisiformis* has been limited, with only one study reported to date (20). In the current study, an overall infection rate of 21.3% was observed, with a significantly higher prevalence in home-raised rabbits (40.2%) than in farm-raised rabbits (1.3%). This difference may be attributed to variations in management systems and feeding practices, with home-raised rabbits reared in less controlled environmental and hygienic conditions, reared in closer contact with dogs (the definitive host), and that did not undergo regular deworming. In contrast, farm-raised rabbits are generally maintained under better sanitary conditions and limited exposure to dogs, resulting in lower infection rates.

Our results align with recent findings reporting a 30% prevalence in domestic rabbits in Egypt and a 23.33% infection rate in local breed rabbits in Iraq, respectively (20, 34). This finding indicates the persistent circulation of this parasite in small-scale or household rabbit-rearing systems. Such findings further emphasize the need to improve hygiene and management practices to reduce transmission risk and control infection.

In contrast, much lower prevalence rates have been recorded in wild rabbits in Europe. In southern Spain, only 2.8% (*n* = 2,923) of wild rabbits were infected (6); in Scotland, a prevalence of 3% (*n* = 786) was reported (35); and in northern Spain, 3.7% (*n* = 267) was detected among lagomorphs (36). Similarly, a Polish study identified a prevalence of 4.74% (*n* = 274) in slaughter rabbits from both small-scale and commercial farms, with no major differences linked to production system or season (11). However, other European studies reported higher prevalence rates in wild rabbits, ranging from 16.1% (*n* = 153) in southern Spain to 33.3% (*n* = 62) in the northeastern region (37). In the Canary Islands, prevalence rates in wild rabbits were found to range between 12% (*n* = 292) and 15% (*n* = 104) (38, 39). A higher average prevalence of 28% (*n* = 95) was also recorded in wild rabbits from England (40).

The lower prevalence in wild populations has been linked to better hygiene practices, such as mandatory antiparasitic treatments for dogs and public awareness campaigns about proper carcass disposal (6). Backyard home-raised rabbits, with more exposure to outdoor environments, appear more vulnerable due to possible contact with feces of definitive hosts such as dogs and foxes (41). Discrepancies in prevalence across studies may be due to differences in numbers of tested animals, populations sampled, sampling design, rabbit type (domestic vs. wild), and the distribution of stray dogs (6, 34). A convenience sampling approach was used in this study based on the ease of accessibility to available samples. This may have introduced potential bias in the prevalence estimates, as such a sample might not accurately reflect the true prevalence of *T. pisiformis* in Egyptian rabbits. Therefore, this represents a limitation of the study. Future research should employ random sampling of both home- and farm-raised rabbits from different Egyptian governorates to allow for more representative and reliable estimates of parasite prevalence. As emphasized in previous research, future studies should focus on identifying other potential definitive or intermediate hosts and understanding the molecular epidemiology of the parasite (6). Another limitation of the study was the inability to analyze the infection status with *T. pisiformis* as a dichotomous outcome in relation to age, management, and season using a logistic regression model. This was due to high collinearity and the small number of available variables, which together prevented us from proceeding with the multivariable analysis.

In this study, positive samples for *T. pisiformis* were more likely to occur in fall and winter compared to those collected in spring. This finding aligns with a previous study that also reported positive cases during the hunting season, spanning from August to March (6). However, in the latter study, sampling was limited to this specific period (i.e., hunting season), making direct seasonal comparisons difficult. A more reliable comparison would require regular sampling throughout the entire year.

It is worth noting that the histopathological evaluation in this study was descriptive rather than semi-quantitative. Although this approach allowed detailed characterization of lesion patterns, the absence of a standardized scoring system represents a limitation that future studies could address to enable more objective comparison of lesion severity.

The present study utilized the mitochondrial *cox1* and *nad1* genes to genetically characterize *T. pisiformis* cysts from Egyptian rabbits and elucidate their phylogenetic relationship with global isolates. These markers were selected for their well-established utility in providing robust species-level resolution and detecting intraspecific variation within cestodes due to their high evolutionary rates and conserved flanking regions (42–44).

Phylogenetic reconstructions using both the neighbor-joining (NJ) and maximum likelihood (ML) methods yielded congruent topologies, consistently placing the Egyptian isolates within the major *T. pisiformis* clade. The strong clustering with sequences from China and Poland highlights a shared genetic ancestry and suggests limited phylogeographic structuring for this parasite on a global scale (43).

The modest sample size (*n* = 4), which is the primary limitation of this study, is important to consider. However, the consistent clustering of all Egyptian isolates from different governorates with global sequences strongly indicates a genetically homogeneous population of *T. pisiformis* in the region. The identified SNPs are therefore more parsimoniously interpreted as natural microvariation within a conserved genetic background rather than as indicators of distinct sub-lineages, a phenomenon previously documented in globally distributed *T. pisiformis* populations (3).

An intriguing finding was the formation of distinct subclusters by sequences derived from wild canids. The fox-derived sequence (AJ239109), while nested within the *T. pisiformis* clade, exhibited a longer branch length and weaker nodal support, suggesting a degree of genetic distance and providing tentative evidence for potential host-associated divergence. It is plausible that the ecological separation and different evolutionary pressures in wild canid hosts may drive subtle genetic structuring within the species, a hypothesis supported by earlier studies (45).

The extensive gene flow facilitated by the wide host range of *T. pisiformis* (45–47) likely maintains the overall genetic homogeneity but may not entirely preclude localized adaptation. As expected, both ML and NJ trees provided clear interspecific differentiation, with other *Taenia* species forming well-supported, distinct clades that corroborate their established taxonomic status.

The present findings underscore a major gap in understanding the economic and public health implications of *T. pisiformis* in Egypt. While local data are scarce, infections elsewhere have been linked to reduced growth performance and carcass value in rabbits. Given the parasite’s life cycle involving dogs, a minor zoonotic risk cannot be excluded. Also, this study confirms the genetic congruence of Egyptian *T. pisiformis* isolates with a globally conserved lineage. While mitochondrial markers (*cox1* and *nad1*) effectively resolved species identity, subtle indications of host-associated variation highlight the need for further investigation with larger sample sizes and advanced genomic tools, such as nuclear markers or full mitogenomes, to better elucidate potential population-level diversity and host adaptation dynamics. In addition, future studies should quantify economic losses and evaluate preventive measures such as regular deworming of farm dogs, improved sanitation, and enhanced health monitoring in rabbit production systems.

## 5 Conclusion

This study highlights the metacestode stage of *T. pisiformis* as a significant parasitic threat to domestic rabbits in Egypt, especially those raised at home. A high prevalence rate, especially during fall and winter in these environments, highlights the consequences of poor management and environmental exposure. Infection rates are further affected by seasonal changes, particularly in the fall and winter, whereas factors such as the age of the rabbits and their geographic location seem to be less influential. Molecular analysis using *cox1* and *nad1* genes confirmed the parasite’s identity and showed genetic diversity among local strains, which closely resembled global isolates. These findings emphasize the urgent need for improved husbandry for comprehensive control measures, including better husbandry practices and regular molecular monitoring, and the integration of molecular tools of *C. pisiformis* in rabbit farming. Furthermore, educating breeders on appropriate carcass disposal methods is essential for public health. Future surveillance initiatives targeting dogs, with the implementation of deworming protocols, are also recommended to disrupt the parasite’s life cycle.

## Data Availability

The datasets presented in this study can be found in online repositories. The names of the repository/repositories and accession number(s) can be found in the article/Supplementary material.
